# Prostate cancer screening in Primary Health Care: the current state of affairs

**DOI:** 10.1186/s40064-015-0819-8

**Published:** 2015-02-13

**Authors:** Weranja KB Ranasinghe, Simon P Kim, Nathan P Papa, Shomik Sengupta, Mark Frydenberg, Damien Bolton, Dimity Pond, Karin Ried, Melanie J Marshall, Raj Persad, Nathan Lawrentschuk

**Affiliations:** Department of Urology, Monash Medical Centre, 823-865 Centre Road, Bentleigh, 3165 Victoria Australia; Yale University; Department of Urology; Cancer Outcomes, Public Policy and Effectiveness Research (COPPER) Center, New Haven, Connecticut USA; University Hospital Case Medical Center, Case Western Reserve University School of Medicine, Urology Institute, Cleveland, Ohio USA; Department of Surgery, Austin Hospital, University of Melbourne, Melbourne, Australia; Department of Surgery, Monash University, Melbourne, Australia; University of Newcastle, Newcastle, Australia; National Institute of Integrative Medicine, Melbourne, Australia; University of New South Wales, NSW (on behalf of PHReNet-GP, a Practice Based Research Network, University of New South Wales), Paddington, Australia; University Hospitals Bristol NHS Trust, Bristol, UK; University of Melbourne, Dept of Surgery, Olivia Newton-John Cancer Research Institute, Austin Hospital and Peter MacCallum Cancer Centre, Dept of Surgical Oncology, Melbourne, Australia

## Abstract

This study aims to examine the current practice of General practitioners (GPs)/primary care physicians in opportunistic screening for prostate cancer (PC) by digital rectal examination(DRE) and Prostate Specific Antigen(PSA) testing and identify any difference in screening practice.

Printed copies and/or electronic versions of a survey was distributed amongst 438 GPs throughout Australia in 2012. Statistical analyses (Wilcoxon rank-sum test, Fisher’s exact test or Pearson chi-square test)were performed by outcomes and GP characteristics.There were a total of 149 responses received (34%), with similar gender distribution in rural and metropolitan settings. 74% GPs believed PSA testing was at least ‘somewhat effective’ in reducing PC mortality with annual PSA screening being conducted by more GPs in the metropolitan setting compared to the rural GPs (35% vs 18.4%), while 25% of rural GPs would not advocate routine PSA screening. When examining the concordance between DRE and PSA testing by gender of GP, the male GPs reported performing PSA testing more frequently than DRE in patients between ages 40 to 69 (p = 0.011).

Urology Society guidelines (77.2%) and College of GPs (73.2%) recommendations for PC screening were thought to be at least ‘somewhat useful’. Although reference ranges for PSA tests were felt to be useful, the majority (65.8%) found it easier to refer to an urologist due to the disagreements in guidelines.

In conclusion, the current guidelines for PSA screening appear to cause more confusion due to their conflicting advice, leaving GPs to formulate their own practice methods, calling for an urgent need for uniform collaborative guidelines.

**Electronic supplementary material:**

The online version of this article (doi:10.1186/s40064-015-0819-8) contains supplementary material, which is available to authorized users.

## Introduction

Australia and New Zealand have the highest incidence of prostate cancer (PC) worldwide with cancer specific five year survival rates exceeding 90% (Ferlay et al. [2010]; [2012]). This high prevalence of PC in Australia is considered to be driven by high rates of opportunistic PSA screening as evidenced by the recent large increases in PSA testing in Australia, with a substantial proportion of detected cancers being lower grade tumours in younger men (Ranasinghe et al. [2014]). At the other end of the age spectrum the recent Concord Health and Ageing in Men Project (CHAMP) study in Australia reported that a significant proportion of men over 70 years were screened for PC (Litchfield et al. [2012]).

Screening for PC using prostate specific antigen (PSA) is highly controversial. Due to the conflicting results from the European Randomized Study of Screening for Prostate Cancer (ERSPC) and the Prostate*,* Lung, Colorectal, and Ovarian (PLCO) Cancer Screening Trial the recent guidelines such as the U.S. Preventive Services Task Force (USPSTF) recommended against the use of PSA screening in all age groups, owing to limited evidence in reducing PC specific mortality and harms attributable to over diagnosis and overtreatment (Schroder et al. [2012]; Andriole et al. [2012]; Chou et al. [2011]). Similarly, in Australia, there are conflicting guidelines from the Urology Society of Australia and New Zealand (USANZ)who advocate the use of DRE and PSA in men between the ages of 50–69 and the General Practice guidelines who do not recommend the routine use of either DRE or PSA in any age group (USANZ [2009]; [RACGP]).

The majority of PSA screening for PC in Australia is conducted by General Practitioners (GPs) in the Primary Care Setting. The lack of clear guidelines may significantly impact clinical practice and negatively influence outcomes for patient’s physicians and healthcare systems. Therefore our aim was to examine the current practice of GPs and Primary Care Physicians in the use of routine DRE and PSA screening as part of opportunistic screening for PC. Furthermore, we aimed to identify any difference in screening practice between GPs by their practice setting (metropolitan and rural) and gender. In addition, we investigated the usefulness of the perceived utility of PSA adjuncts such as age PSA ranges, median PSA, prostate health index (PHI) (Stephan et al. [2014]) in the Primary Care Setting.

## Methods

### Study population

After ethics approval, a survey reviewing PSA screening was distributed amongst 438 GPs throughout Australia. Printed copies of the questionnaire and a web link to an online questionnaire were distributed using local GP Practice Based Research Networks via the National Institute of Integrative Medicine, Victoria (n = 130), Centre for Primary Health Care and Equity, NSW (n = 52) and the University of Newcastle, NSW(n = 136) in 2012. When mailed out, a reply paid envelope was included and three reminders were mailed out post initial survey. In addition, 120 questionnaires were distributed at the UroGP conference (University of Melbourne, Austin Hospital, Melbourne Australia), a local GP’s meeting in Melbourne. No incentives were provided for any of the respondents.

### Questionnaire

This was developed in the USA to assess the perceptions and attitudes of PSA screening and treatment in an average risk man (a man with no significant co-morbidities, no family history (FHx) of PC and >10 yrs life expectancy) and piloted amongst a small number of GPs (n = 10) in Australia (Additional file 1). Ethics approval was sought from the Ethics and Research Committee at the Alfred Hospital.

The question items on the survey included the characteristics of the respondents such as age group, location of practice (metropolitan, regional or rural), gender and race. The effectiveness of PC screening modalities PSA and DRE as well as radical prostatectomy, radiotherapy and active surveillance in reducing cancer mortality due to prostate cancer in an average risk man was assessed.

The respondents were also asked about the recommendation of utilisation of PSA screening and DRE as the “best practice” in an average risk man for the age groups of 40–69 yrs, 50 to 59 yrs, 70–75 yrs and >75 years and the appropriate frequency of screening. In addition, the influence of the recommendations regarding PC screening from the leading organisations and the perceived utility of the effectiveness of reference ranges of median PSA, age-related PSA range, free to total PSA and the utilisation of prostate health index were also assessed.

### Statistical analysis

Comparisons by gender and location (metropolitan versus rural based GPs) were made with the Wilcoxon rank-sum test for ordinal response outcomes and the Fisher’s exact test or Pearson chi-square test for binary response outcomes. Testing the equality of intervals of PSA and DRE testing was performed with the Wilcoxon matched-pairs signed-ranks test. Percentages quoted in the text include participants who did not answer the specified question, unless otherwise noted. All tests were two-sided with a significance level set at <0.05. Analysis was performed with Stata v.12.0 SE (Statacorp, College Station, TX).

### Ethical standards

Ethics approval was sought from the Ethics and Research Committee at the Alfred Hospital.

## Results

### Characteristics of respondents

There were a total of 149 responses received (34%), the majority (65.1%) were UroGP conference attendees (Table 1). Of the total respondents 68.5% were males, worked in a metropolitan setting (62.4%) and were from the state of Victoria (49%). 38.9% of the respondents were between the ages of 50 to 59 and the mean number of years in practice was 26.2 (median 27.5 years; range 1–55 years). The distribution of gender was almost identical in metropolitan and rural areas (female GPs in metropolitan regions 29.7%, in rural areas 29.6%). Detailed characteristics are demonstrated in Table 1.

**Table 1:** **Characteristics of respondents**

Characteristic of respondent	Number of respondents (percentage)
**Gender**	
Male	102 (68.5%)
Female	43 (28.9%)
Not answered	4 (2.7%)
Area of practice*	
Metropolitan	93 (62.4%)
Combined rural	55 (36.9%)
Regional	34 (22.8%)
Rural	21 (14.1%)
Not answered	1 (0.7%)
**Age**	
≤40	21 (14.1%)
41 to 49	23 (15.4%)
50 to 59	58 (38.9%)
60 to 69	34 (22.8%)
≥70	11 (7.4%)
Not answered	2 (1.3%)
**Years in practice**	
<10 years	18 (12.1%)
10 – 20 years	30 (20.1%)
>20 years	96 (64.4%)
Not answered	5 (3.4%)
**State**	
Victoria	73 (49.0%)
New South Wales	37 (24.8%)
Queensland	13 (8.7%)
Western Australia	9 (6.0%)
South Australia	9 (6.0%)
Other/Not answered	8 (5.4%)
**Race**	
Asian	30 (20.1%)
Caucasian	107 (71.8%)
Other/Not answered	12 (8.1%)
**Breakdown of responses by recruitment**	
UroGP conference	97/120 (80.8%)
National Institute of Integrative Medicine, Victoria	17/130 (13.1%)
Centre for Primary Health Care and Equity, NSW	14/52 (26.9%)
University of Newcastle, NSW	21/136 (15.4%)

*Due to small sample size, GPs working in the regional and rural sectors were combined and labelled as rural practice in the analysis.

### Beliefs in current therapy

**Figure 1:**
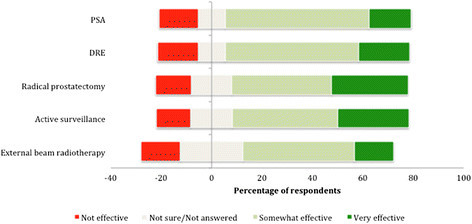
**Beliefs in current therapy for the management of PC in Australia.**

Seventy four percent of GPs believed that PSA testing was at least ‘somewhat effective’ in reducing PC mortality in an average risk male with similar results for digital rectal examinations (DRE). In terms of treatment, 70% of GPs thought that radical prostatectomy and active surveillance were at least somewhat effective in reducing PC mortality in an average risk man. External beam radiotherapy was thought to be effective by 59% of respondents, though there were a relatively high number of “not sure” respondents for this modality (Figure 1).

For active surveillance, there was a difference in perceived effectiveness by gender (female GPs) and by practice setting (Metropolitan) compared to their peers (Table 2).

**Table 2:** **Perceived effectiveness of active surveillance by gender of GP and by setting of practice**

	Very effective	Somewhat effective	Not effective	P value	Not sure/not answered
**Gender**				0.011	27
Female GP (%)	17 (44.7)	20 (52.6)	1 (2.6)		
Male GP (%)	24 (28.6)	42 (50.0)	18 (21.4)		
**Setting**				0.038	26
Metro GP (%)	32 (40.0)	37 (46.3)	11 (13.8)		
Rural GP	9 (20.9)	25 (58.1)	9 (20.9)		

Percentages and p-values calculated only for subjects that gave a response.

### Current practice of DRE and PSA

There were no significant differences in DRE based screening by GP gender or practice setting for men between 40 to 69 years of age (Table 3). However, there were significant differences in frequency of PSA screening by practice setting but not by GP gender (Table 4). Furthermore, when examining the concordance between DRE and PSA testing by GP gender, male GPs reported performing PSA testing more frequently than DRE in patients aged 40 to 69 (p = 0.011) while female GPs practised similar interval times between tests (p = 0.608) (Table 4a/b). These results were similar for patients between ages 50 to 59; male GPs (p = 0.002), female GPs (p = 0.739). The concordance between frequencies of DRE and PSA screening are seen in the Additional file 2: Table S1(a) and (b).

**Table 3:** **Frequency of DRE and PSA based screening by location of practice and gender for patients aged 40 to 69**

	Every year	Every 2 years	Every 5 years	Would not recommend	P value	Other/not answered
**DRE**						
**Setting**					0.194	
Metro GP (%)	25 (34.3)	25 (34.3)	8 (11.0)	15 (20.5)		20
Rural GP	6 (22.2)	8 (29.6)	7 (25.9)	6 (22.2)		28
**Gender**					0.388	
Female GP (%)	10 (32.3)	12 (38.7)	5 (16.1)	4 (12.9)		12
Male GP (%)	21 (31.3)	19 (28.4)	10 (14.9)	17 (25.4)		35
**PSA**						
**Setting**					0.006	
Metro GP (%)	35 (49.3)	15 (21.1)	7 (9.9)	14 (19.7)		22
Rural GP (%)	7 (18.4)	14 (36.8)	4 (10.5)	13 (34.2)		17
**Gender**					0.434	
Female GP (%)	10 (30.3)	9 (27.3)	7 (21.2)	7 (21.2)		10
Male GP (%)	31 (41.9)	19 (25.7)	4 (5.4)	20 (27.0)		28

Percentages and p-values calculated only for subjects that gave a response.

**Table 4:** **Frequency of PSA screening by gender and location of practice for patients aged 40 to 69 (a) and patients aged 50 to 59 (b)**

	Every year	Every 2 years	Every 5 years	Would not recommend	P value	Other/not answered
**4(a)**						
*In patients aged 40 to 69:*						
**Setting**						
Metro GP (%)	35 (49.3)	15 (21.1)	7 (9.9)	14 (19.7)	0.006	22
Rural GP (%)	7 (18.4)	14 (36.8)	4 (10.5)	13 (34.2)		17
**Gender**						
Female GP (%)	10 (30.3)	9 (27.3)	7 (21.2)	7 (21.2)	0.434	10
Male GP (%)	31 (41.9)	19 (25.7)	4 (5.4)	20 (27.0)		28
**4(b)**						
*In patients aged 50 to 59:*						
**Setting**						
Metro GP (%)	48 (64.0)	17 (22.7)	3 (4.0)	7 (9.3)	0.002	18
Rural GP (%)	16 (36.4)	15 (34.1)	2 (4.6)	11 (25.0)		11
**Gender**						
Female GP (%)	18 (52.9)	8 (23.5)	4 (11.8)	4 (11.8)	0.942	9
Male GP (%)	44 (53.0)	24 (28.9)	1 (1.2)	14 (16.9)		19

Percentages and p-values calculated only for subjects that gave a response.

### Influential organisations

The majority of the GPs surveyed thought that USANZ (77.2%) and the College of GPs (73.2%) recommendations were at least ‘somewhat influential’. There were no significant differences in gender or practice setting when comparing which guidelines were the most useful.

Interestingly, just over half (51.0%) of GPs disagreed with the USPSTF recommendations against PSA and DRE for screening in asymptomatic men.

### Types of PSA testing

Of the PSA tests available, the majority of GPs believed that age-related PSA ranges (73.2%) and free-to-total PSA ratio (78.5%) were effective, whilst almost half (40.3%) of the surveyed GPs thought that the median PSA level provided was not. Only 16.1% of GPs had heard of the PHI and of these only 20.8% used it in clinical practice.

Furthermore, 61.1% of GPs thought that 5-alpha-reductase-inhibitors would affect the PSA test, whilst 32.2% were unsure of the effect or did not provide a response. Only 6.7% of GPs believed the drugs had no effect on a patient’s PSA level.

### Referral to an urologist

Nearly two thirds (65.8%) of GPs found it easier to refer to an urologist if there was any confusion regarding results due to the current screening guidelines. There was a significant difference in willingness to refer by GP gender with female GPs more likely to refer (87.5% vs. 65.6%, p = 0.011; Table 5). There was also a difference observed in metropolitan vs. non-metropolitan (76.7% vs. 63.3%, p = 0.094) though this did not reach significance.

**Table 5:** **Referral to an urologist by gender and location of practice**

	Refer	Not refer	P value	Not answered
**Setting**				
Metro GP	66 (76.7)	20 (23.3)	0.094^#^	7
Rural GP	31 (63.3)	18 (36.7)		6
**Gender**				
Female GP (%)	35 (87.5)	5 (12.5)	0.011*	3
Male GP (%)	61 (65.6)	32 (34.4)		9

*Fisher’s exact test; ^#^Pearson chi-square.

Percentages and p-values calculated only for subjects that gave a response.

## Discussion

Screening for PC remains highly controversial due to the lack of consensus from clinical practice guidelines. Thus, some guidelines advocate regular screening for PC, whilst other established bodies such as the USPSTF do not recommend screening in any age group (Schroder et al. [2012]; Andriole et al. [2012]; Chou et al. [2011]), leading to decrease in PC screening in the USA (Aslani et al. [2013]). In Australia, the leading bodies have completely opposing guidelines. The USANZ policy in 2009 recommended starting a single PSA test and DRE at 40 years whilst the college of GP’s do not recommend PSA screening or DRE unless the patient specifically requests it and is clearly counselled (USANZ [2009]; [RACGP]).Thus a clear message from a peak body is still lacking. Our results show that as similar proportions of GPs take into account guidelines from all the major bodies, causing confusion not only on the frequency of PSA screening but also whether to conduct DRE and their appropriate frequency. These conflicts in existing guidelines directly translate in to significant variations in clinical practice, leaving GPs to formulate their own practice methods without any clear guidelines.

In this situation where there are conflicting guidelines, the decision to screen is strongly biased by Primary Health Care providers views, rather than those of the patients, as the latter usually have inadequate knowledge to make an informed decision (Hoffman et al. [2009]; Han et al. [2013]). Our study demonstrates higher rates of PSA screening in asymptomatic men compared to another Danish study (Jessen et al. [2013]). Certainly GPs views may be influenced by medical training. Marcella et al. demonstrated that during medical training, students generally are very optimistic about the benefits of screening for PC but with increased knowledge, a more conservative view of screening is adopted (Marcella et al. [2007]). While further refinement/consistency of teaching to medical students regarding other issues such as family history in PC may help case selection, our findings clearly highlight the need for more structured collaborative guidelines to help inform GPs well as their patients.

The process of screening should be by promoting informed decision making taking in to account the patient’s concerns, as some men fear impotence and incontinence after treatment if diagnosed with cancer (Parchment [2004]). Cultural sensitivity must also be taken into account (Chan et al. [2003]). Interestingly, one study demonstrated, that when men were provided with sufficient information, they were less likely to accept a recommendation by a GP to undergo PSA screening (Gattellari & Ward [2005]). However, the physician’s positive engagement in shared decision-making, tailored social influences promoting PC prevention among certain cultures, as well as institutional screening policy, has the potential to increase early detection and reduce morbidity (Woods et al. [2006]).

An intriguing finding in this study was that younger GPs tend to conduct more regular PSA testing rather than DRE. An abnormal DRE is sensitive in detecting PC but when combined with a an abnormal serum PSA sensitivity is further increased (95%, PPV = 62%) (Martinez De Hurtado et al. [1995]). However, there are a number of possibilities as to why DRE is not utilised as readily as a PSA test. The findings of DRE has potentially implied subjectivity especially when not performed regularly, due to difficulty palpating the whole prostate (Koulikov et al. [2012]) and limited urology skills as a medical student (Kaplan et al. [2012]). In addition patient factors such as cultural attitudes to DRE (Consedine et al. [2006]) and embarrassment of the patient (Parchment [2004]) may also play a role.

PSA is a non-specific test and can be elevated in a number of benign and inflammatory conditions. Therefore many other methods such as age PSA ranges, median PSA, PHI have been developed and advocated to enhance the accuracy of detecting PCs (Stephan et al. [2014]). However, no single test is currently accepted as the best modality and should be interpreted with using judicious clinical acumen and individualised to the patient. Furthermore, the USANZ policy recommends taking into account family history, ethnicity, DRE and PSA derivatives such as PSA velocity and free-total PSA ratio when screening for PC (USANZ [2009]).Thus some of these ranges, if not all, are routinely included in the generated report by pathology laboratories and these results are provided to the referring GP. Our results suggest that the introduction of these tests and inclusion of these ranges are likely to cause more confusion rather than being a helpful guide to GPs and could possibly lead to more referrals to the urologists which could even prompt earlier biopsies.

There was a significant discrepancy in PC screening practice between practice settings, with lower rates of screening evident in rural areas. This is in keeping with lower PSA screening and radical prostatectomy rates leading to poorer survival and mortality outcomes in PC for males living in rural Australia when compared to their urban counterparts (Baade et al. [2011]). These findings are largely influenced by limited access to resources for routine screening and the lack of locally available urology services, a predicament also faced in other countries (Baldwin et al. [2013]; Patel et al. [2010]). The role of the poorly accessible urological service cannot be underestimated as this may deter rural GPs from screening asymptomatic men as readily and adopt their practice taking into account these limitations in resources. In addition, the conflict with current screening guidelines and the possibility that metropolitan males may request PSA screening more readily than their rural counterparts, which may further influence the decision to screen urban men more readily.

### Limitations

The effect that gender and practice location seen in our study are unlikely to have influenced each other, due to similar distributions of respondents in each group. One drawback in our study is the low response rate despite efforts to increase the response rate such as reminders (both postal and email) and reply paid postage envelopes. The non-respondents characteristics were not readily available as the questionnaire was completely anonymous. A further limitation of this study is that the majority of the responses came from a selected group of GPs who attended the UROGP conference and were aware of issues in PC, which may have introduced some selection bias in to the study. In addition there was a skew towards an older GP population answering the survey.

We did not test the GPs attitudes towards the risks downstream of PSA testing. In Australia as a high proportion of men with low risk prostate cancer undergo active surveillance (Evans et al. [2013]), the discussions regarding treatment and diagnosis have to be separated due to the difficulty of estimating likely mortality benefits without a biopsy and as such downstream effects such as incontinence and ED need to be discussed once the decision has been reached that treatment is actually required.

In conclusion, our study demonstrates that in the absence of clear guidelines from a peak body, there is significant variation in current practice in screening for PC. The conflicting messages given out in these guidelines appear to be causing more confusion rather than providing guidance, leaving GPs to formulate their own practice methods. These findings call for an urgent need for uniform guidelines for PC screening practices amongst GPs which should be formed as collaborative effort by GP continuing medical education (CME) bodies and all specialities involved in the care of PC. This should be then followed by a robust education campaign aimed at GPs as well as individuals. As such, the recent National Health and Medical Research Council (NHMRC) have attempted to overcome these variations by publishing recent guidelines (NHMRC [2014]). However, the practical application of these guidelines in day to day practice by the GPs is yet to be determined.

## Additional files

## Electronic supplementary material

Additional file 1: Sample of the questionnaire distributed amongst the GPs. (PDF 250 KB)

Additional file 2: Table S1.: Demonstrates the concordance between frequency of PSA testing and DRE. (DOC 36 KB)

Below are the links to the authors’ original submitted files for images.

Authors’ original file for figure 1
