# Supplementary material for: Prostate cancer screening in Primary Health Care: the current state of affairs
Source: Springerplus. 2015 Feb 13;4(1):78. doi: 10.1186/s40064-015-0819-8 (PMC4332913; doi:10.1186/s40064-015-0819-8)
Supplement: Supplementary file 1 — Additional file 1: Sample of the questionnaire distributed amongst the GPs. (PDF 250 KB) [file 40064_2015_819_MOESM1_ESM.pdf]

Current practice in screening for prostate cancer in primary care: A

1. What is your gender?

- Female
- Male

2. What is your age?

- Less than 40 years
- 41 to 49 years
- 50 to 59 years
- 60 to 69 years
- More than 70 years

3. How many years have you been in practice?

Years in practice:

4. How would you classify your race?(Check one.)

- Asian or Asian American
- Black or African-American
- Hispanic or Latino
- White or Caucasian
- Other

5. Which state is your practice located?

	Metropolitan	Regional	Rural
Victoria			
New South Wales			
ACT			
Western Australia			
NT			
South Australia			
Queensland			
Tasmania			
New Zealand			
UK			

Current practice in screening for prostate cancer in primary care: A

Please answer the following items about prostate cancer screening and guide...

Assume an average risk man is a patient without any significant comorbidity or family history of prostate cancer, and a greater than 10 year life expectancy.

1. How effective do you believe the following screening tests and primary therapies are in reducing cancer mortality due to prostate cancer in an average risk man? (Please check one box on each line.)

	Very effective	Somewhat effective	Not effective	Not sure
PROSTATE-SPECIFIC ANTIGEN (PSA)	○	○	○	○
DIGITAL RECTAL EXAM (DRE)	○	○	○	○
RADICAL PROSTATECTOMY	○	○	○	○
EXTERNAL BEAM RADIATION (EBT)	○	○	○	○
ACTIVE SURVEILLANCE	○	○	○	○

2. To what extent does your practice recommend the following screening test as the "best practice" for the following age groups of an average risk man? Please indicate for each age group.
DIGITAL RECTAL EXAM (DRE)

	Every year	Every 2 years	Every 5 years	Would not recommend	Other
40 to 69 years	○	○	○	○	○
50 to 59 years	○	○	○	○	○
70 to 75 years	○	○	○	○	○
More than 75 years	○	○	○	○	○

Other (please specify)

3. To what extent does your practice recommend the following screening test as the "best practice" for the following age groups of an average risk man? Please indicate for each age group.
PROSTATE-SPECIFIC ANTIGEN (PSA)

	Every year	Every 2 years	Every 5 years	Would not recommend	Other
40 to 69 years	○	○	○	○	○
50 to 59 years	○	○	○	○	○
70 to 75 years	○	○	○	○	○
More than 75 years	○	○	○	○	○

Other (please specify)

4. How influential are the recommendations regarding prostate cancer screening from the following organizations on the content of your residency education curriculum on this topic? (Please check one box on each line.)

	Very influential	Somewhat influential	Not influential	Don't know
Urology Society of Australia and New Zealand	○	○	○	○
Royal College of Surgeons	○	○	○	○
Royal Australasian College of General Practitioners	○	○	○	○
U.S. Preventive Services Task Force	○	○	○	○
American Cancer Society	○	○	○	○
European Urological Association Guidelines	○	○	○	○

5. The U.S. Preventive Services Task Force recently changed their recommendations to against the routine use of Prostate-Specific Antigen (PSA) and Digital Rectal Exam (DRE) for prostate cancer screening in asymptomatic men. To what extent to you agree or disagree with this new recommendation?

- ○ Strongly agree
- ○ Somewhat agree
- ○ Somewhat disagree
- ○ Strongly disagree
- ○ Don't know

6. How effective do you believe the following screening test parameters provided by the pathologist as references are (usually in brackets next to the PSA result or below the test as an addendum)? (Please check one box on each line.)

	Effective	Not effective
The median PSA level provided	○	○
Age-related ranges of PSA	○	○
The Free: total PSA ratio	○	○

7. Are you aware of a recently introduced blood test called the Prostate Health Index?

- ☐ Yes
- ☐ No

8. If 'Yes' to Question 12, do you utilize it?

- ○ Yes
- ○ No

9. Due to all the confusion is it easier simply to refer to a urologists if there is any doubt about a result?

- Yes
- No

10. With the new drug for BPH treatment dutasteride now available and the older drug finasteride(both 5-alpha reductase inhibitors) do you believe such agents may have an impact on a patient's PSA?

- Yes
- No
- Unsure

11. At what PSA level would you recommend a urology referral?

- Less than 2
- Between 2 and 5
- Between 5 and 10
- Greater than 10
