# Supplementary material for: Prostate cancer screening in Primary Health Care: the current state of affairs
Source: Springerplus. 2015 Feb 13;4(1):78. doi: 10.1186/s40064-015-0819-8 (PMC4332913; doi:10.1186/s40064-015-0819-8)
Supplement: Supplementary file 2 — Additional file 2: Table S1.: Demonstrates the concordance between frequency of PSA testing and DRE. (DOC 36 KB) [file 40064_2015_819_MOESM2_ESM.doc]

**Table S1: Demonstrates the concordance between frequency of PSA testing and DRE. PSA is on the horizontal axis, DRE on the vertical. Red fields are where PSA is practised more frequently than DRE. Blue fields are the reverse.**

a) Prostate cancer screening practice of male GPs in patients aged 40 to 69

| PSA  DRE | Every year | Every 2 years | Every 5 years | Would not recommend |
| --- | --- | --- | --- | --- |
| Every year | 16 | 3 | 0 | 1 |
| Every 2 years | 7 | 7 | 1 | 0 |
| Every 5 years | 3 | 3 | 1 | 1 |
| Not recommend | 3 | 2 | 0 | 11 |

p=0.011, 43“Other” or no response for either question

(b) Prostate cancer screening practice of female GPs in patients aged 40 to 69:

| PSA  DRE | Every year | Every 2 years | Every 5 years | Would not recommend |
| --- | --- | --- | --- | --- |
| Every year | 8 | 0 | 1 | 0 |
| Every 2 years | 2 | 8 | 1 | 1 |
| Every 5 years | 0 | 0 | 5 | 0 |
| Not recommend | 0 | 0 | 0 | 3 |

p=0.608, 14“Other” or no response for either question
