# Supplementary figures and images for: Prostate cancer screening in Primary Health Care: the current state of affairs
Source: Springerplus. 2015 Feb 13;4(1):78. doi: 10.1186/s40064-015-0819-8 (PMC4332913; doi:10.1186/s40064-015-0819-8)

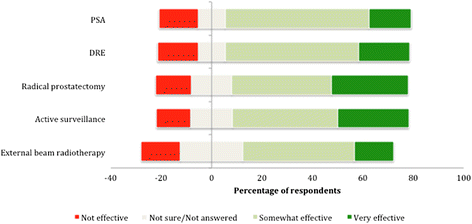

Supplement: Supplementary file 3 — Authors’ original file for figure 1 [file 40064_2015_819_MOESM3_ESM.gif]
